# Chemical composition, enantiomeric analysis and anticholinesterase activity of *Lepechinia betonicifolia* essential oil from Ecuador

**DOI:** 10.1080/13880209.2021.2025254

**Published:** 2022-01-20

**Authors:** James Calva, Luis Cartuche, Salomé González, José Vinicio Montesinos, Vladimir Morocho

**Affiliations:** Departamento de Química, Universidad Técnica Particular de Loja, Loja, Ecuador

**Keywords:** Enantiomeric distribution, chemical profiling, acetylcholinesterase, pinene, sabinene

## Abstract

**Context:**

Due to the interesting potential of essential oils (EO) against cholinesterases and their close relation in Alzheimer’s disease, the EO of *Lepechinia betonicifolia* (Lam) Epling (Lamiaceae), a native shrub from Ecuador, was assessed. Chemical profiling and enantiomeric distribution were also recorded for the first time.

**Objective:**

To analyse the chemical profile including the enantiomeric composition and anticholinesterase effect exerted by EO of *L. betonicifolia*.

**Materials and methods:**

The EO of *L. betonicifolia* fresh aerial parts was obtained by hydrodistillation in a Clevenger-type apparatus. Physical properties were determined according to standard norms. The chemical composition was determined by GC-MS and GC-FID. Enantioselective GC-MS analysis was carried out by using a capillary chiral column. Anticholinesterase effect was assessed by Ellman’s method with acetylthiocoline as substrate and Ellman’s reagent (DTNB) to detect its hydrolysis at 405 nm for 60 min. Donepezil was used as a reference drug. EO was dissolved in methanol to reach 10 mg/mL concentration and two more 10× dilutions were included.

**Results:**

Thirty-nine constituents were identified corresponding to 97.55% of the total oil composition. The main components were β-pinene (30.45%), sabinene (27.98%), α-pinene (4.97%), β-phellandrene (4.79%), *E*-caryophyllene (4.44%) and limonene (3.84%). *L. betonicifolia* EO exerted a strong inhibitory effect over the AChE enzyme with an IC_50_ value of 74.97 ± 1.17 μg/mL.

**Discussion and conclusions:**

Current chemical characterisation and anticholinesterase effect of EO of *L*. *betonicifolia* encourage us to propose this EO as a candidate for the preparation of functional foods or as adjuvant therapy for Alzheimer’s disease.

## Introduction

Essential oils (EO) are a mixture of complex volatile compounds, synthesised by various parts of the plant (Akthar et al. 2014), they have an aromatic nature due to a mixture of multiple chemical substances, including terpenes, aldehydes, alcohols, esters, phenolics, ethers, and ketones (Degenhardt et al. 2009). The chemical composition of an EO can vary from species to species and even due to different ecological conditions and is responsible for a plant’s sensorial and biological properties (Perrino et al. 2021). Variation also depends on the part collected and employed for extraction as well as the mode of culturing (Santo-Gómes and Fernandes-Ferreria 2001), ecotypes (subpopulations genetically differentiated) (Barra 2009), management conditions as post-harvesting, processing and extraction method that can affect the types and amounts of compounds detected (Zhang and Guo 2020). Furthermore, several components in an EO are chiral compounds, often occurring as pure enantiomers or in an enantiomeric excess and, seldom, in racemic form (Finefield et al. 2012).

*Lepechinia* is a genus of plants in the Lamiaceae family and consists of ca. 43 species (Camina et al. 2018). It is a diverse and widespread genus important in indigenous cultures for its medicinal and antiseptic properties. Their distinctive odour given by their chemical composition can also be responsible for their medicinal effects (Drew and Sytsma 2013). Most of the *Lepechinia* species in South America are distributed between 1500 and 4000 m a.s.l., within a wide range of habitats. Most of them occur in relatively dry open habitats of the Andean highlands (Drew and Sytsma 2013). In Ecuador, only 9 native and 4 endemic species are reported. *Lepechinia betonicifolia*, is a native shrub from the Andes, found between 1000 and 3000 m a.s.l., and distributed in the provinces of Azuay, Imbabura, Loja, Pichincha, and Tungurahua (Jørgensen and León-Yánez 1999). *L. betonicifolia*, commonly known as Matico or salvia real, has been used for the treatment of bruises, inflammation and wound infections. The whole plant, in infusion or sitz baths, is used to treat skin conditions as bruises as well as a cicatrising agent and for intestinal ulcers (De la Torre et al. 2008). Most of the *Lepechinia* species are reported for having antiseptic properties and in folk medicine are used for the treatment of uterine tumours, stomachaches, diabetes and diarrhoea (Ramírez et al. 2018). No studies about ethnomedical uses on neurodegenerative disease have been reported for this species but, the effectiveness of essential oils as anti-inflammatory, antioxidants, and inhibitors of cholinesterase have been well documented, so, our research group is continuously investigating the properties of species of *Lepechinia*.

Several studies on the *Lepechinia* genus reported heterogeneity of identified compounds, which has not allowed the establishment of a typical metabolic pattern. So far to date, the essential oils of some Lamiaceae, including two species belonging to the genus *Lepechinia* spp. have been studied (Velasco-Negueruela et al. 1994; Cicció et al. 1999; Acevedo et al. 2005; Borges et al. 2006; Arze et al. 2009; Valarezo et al. 2012; Panamito et al. 2021). Two related studies of EO from *L. mutica* (Benth) Epling, reported a great variation in the chemical composition. Malagón et al. (2003) identified 54 compounds, among them β-phellandrene (30%), camphene (13%), limonene (8%), 3-carene (6%) and α-pinene (6%) were the most abundant. On the other hand, Ramírez et al. (2018) reported the identification of δ-3-carene (24.23%), eudesm-7(11)-en-4-ol (13.02%), thujopsan-2-α-ol (11.90%), β-pinene (7.96%), valerianol (5.19%), and co-eluting limonene and β-phellandrene (4.47%) as the main constituents. In another study, with a related species from the same geographical location, Gilardoni et al. (2018) reported from *L. heteromorpha* (Briq) Epling, the identification of 25 constituents, where viridiflorene (27.3%), (*E*,*E*)-α-farnesene (1.4%), ledol (21.2%), spirolepechinene and (*E*)-β-caryophyllene (7.1%), allo-aromadendrene (6.1%) were the main constituents.

A few studies about anticholinesterase potential have been reported to date. A recent study from the EO of *L*. *paniculata* (Kunth) Epling showed an interesting selective inhibitory activity against acetylcholinesterase (AChE) and butyrylcholinesterase (BuChE) enzymes, with IC_50_ values of 28.2 ± 1.82 and 28.8 ± 1.5 µg/mL, respectively (Panamito et al. 2021). In the present work, we analysed the chemical profiling and the enantiomeric distribution by using gas chromatography coupled to mass spectrometry (GC-MS) and gas chromatography coupled to flame ionisation detector (GC-FID), and finally, we assessed the anticholinesterase inhibitory capacity of the EO extracted from the aerial parts of *L. betonicifolia*. Several species of *Lepechinia* have been investigated for their biological activities in our research group to validate the ethnomedical uses attributed to this genus and the aim of our study was to preliminarily characterise the potential of this species as a cholinesterase inhibitor. With this basis, we will propose later a complete analysis of the kinetic mechanisms against AChE of the pure major enantiomers found in the EO as well as, the inhibition profile of AChE isolated from brain rats or other related sources to validate the *in vitro* results.

## Materials and methods

### Collection of plant material

Fresh aerial parts of *L. betonicifolia* were collected during the flowering state in May 2017 in the locality of “Cerro Gañil,” Saraguro, in the province of Loja (coordinates 3°36′46ʺS and 79°19′3ʺ W), at an altitude of 2800 m a.s.l. The species was identified by Dr. Nixon Cumbicus, curator of the herbarium of Universidad Técnica Particular de Loja, identified with voucher number HUTPL13794 and collected under permission from the Ministry of Environment of Ecuador (MAE-DNB-CM-2016-0048).

### Extraction of essential oil

The aerial parts were subjected to hydrodistillation for 4 h using Clevenger-type equipment. The distillation process was repeated 4 times. Anhydrous Na_2_SO_4_ was added to the flask containing the EO with the moisture of the process of distillation to remove it and then the EO was stored at 4 °C for subsequent experiments.

### Physical properties

Three physical properties of EO were determined. Relative density (d20) was determined according to the international standard AFNOR NF T75-111 (ISO 279:1998) in an analytical balance (Mettler AC100 model) and 1 mL pycnometer, the refractive index was performed in an ABBE refractometer (Boeco, Germany) according to the international standard AFNOR NF 75-112 (ISO 280:1998), and specific optical rotation was determined in a Hanon P 810 automatic polarimeter according to the standard ISO 592:1998. Measurements were performed three times at 20 °C.

### Gas chromatography – mass spectrometry

The GC analysis of *L. betonicifolia* EO was performed with an Agilent 6890 N gas chromatography coupled to a 5973 N mass spectrometer (Santa Clara, CA, USA) instrument equipped with a DB-5MS capillary column (5%-phenyl-methyl polysiloxane, 30 m × 0.25 mm i.d., 0.25 μm film thickness; J & W Scientific, Folsom, CA, USA), working with the following temperature program: 5 min at 60 °C then 3 °C/min up to 165 °C, then 15 °C/min up to 250 °C held for 10 min. The injector and detector temperatures were: 220 °C; carrier gas: Helium; flow rate: 1 mL/min; split ratio: 1:40; acquisition mass range: 40–350 *m/z*; and electron-impact mode (EI, 70 eV). The samples were diluted at 1:100 in dichloromethane (Fisher Scientific, 99.9% purity) and 1 µL of the solution was injected (Calva et al. 2017).

Further identification of constituents was based on a comparison of their linear retention indices with those reported in the literature (Adams 2007). The LRI of Van Den Dool and Kratz were calculated using retention times according to the retention times of a homologous series of C9-C25 (Fluka purity 99%) injected under the same conditions as the essential oil (Van del Dool and Kratz 1963).

### Gas chromatography flame ionisation detector

Quantitative analysis of the essential oil was performed on an Agilent gas chromatograph (model 6890 N) coupled to a flame ionisation detector (FID) and using a 7683 series autoinjector (Agilent, Little Falls, DE, USA). The analytical parameters were the same as the GC-MS analysis. Component relative percentages were calculated based on GC peak areas without using correction factors (Calva et al. 2019).

### Enantioselective analysis

Enantioselective GC-MS analysis of the EO of *L. betonicifolia* aerial parts was accomplished with a GC-MS system (Agilent Technologies) using a chiral capillary column, diethyl tertbutylsilyl-β-cyclodextrin (25 m × 0.25 mm film thickness 0.25 μm) from Mega (Legnano, MI, Italy). Working with the following program: 60 °C for 2 min, and then increased to 220 °C, with a gradient rate of 2 °C/min, held for 2 min. The mass spectrometer operated in electron impact ionisation mode at 70 eV, with a mass range of *m/z* 40–350 full scan mode. The ion source temperature was set at 220 °C. Helium was the carrier gas at a flow rate of 1.0 mL/min. The injector operated in split mode (40:1) at 200 °C. The samples were diluted at 1:100 in dichloromethane (Fisher Scientific, 99.9% purity), and then 1 µL was injected. Further identification of constituents was based on comparison with enantiomerically pure standards.

### Acetylcholinesterase inhibitory activity

The inhibition of the AChE enzyme was measured using the spectrophotometric method developed by Ellman et al. (1961), with slight modifications as suggested by Rhee et al. (2001) and Valarezo et al. (2021). Acetylthiocholine iodide (ATCh) was used as the substrate and different concentrations of EO in methanol to detect the inhibition of AChE. The reaction mixture contained 40 μL of Buffer Tris (Tris–HCl, 50 mM, pH 8 adjusted with 2 N HCl, 0.1 M of NaCl and 0.02 M of MgCl_2_·6H_2_O), 20 μL of the tested sample solution, 20 μL of ATCh (15 mM, PBS pH 7.4), and 100 μL of DTNB (3 mM, dissolved in Buffer C). Pre-incubation was carried out for 3 min at 25 °C and continuous shaking. Finally, the enzymatic reaction was started with the addition of 20 μL of 0.5 U/mL AChE (137 U/mg solid) and, the amount of product released was monitored at 405 nm at 25 °C for 60 min in an EPOCH 2 (BIOTEK®) microplate reader every 1 min.

All reactions were performed in triplicate in a 96-well flat-bottom microplate. Tested sample solutions from the essential oil were made by dissolving an aliquot of EO (according to its density) to obtain an initial concentration of 10 mg in 1 mL MeOH. Two more dilutions (10× factor dilution) were included to obtain final concentrations of 1000, 100, and 10 μg/mL. The IC_50_ value was calculated by curve fitting of data (non-linear regression analysis, PRISM 8.0.1, GraphPad, San Diego, CA, USA). Donepezil was used as a positive control with an IC_50_ value of 13.80 ± 1.01 nM (McGleenon et al. 1999; Valarezo et al. 2021). Any increase in absorbance because of spontaneous hydrolysis of ATCh was corrected by subtracting the absorbance at the end of pre-incubation from the absorbance measured after the addition of the enzyme.

## Results and discussion

The EO obtained through hydro distillation from the aerial parts of *L*. *betonicifolia* presented a yellow colour, a yield of 0.17 ± 0.03% (w/w) and a total volume of 16.5 g of EO from 9.85 kg of plant material. The density of EO was 0.87 ± 0.01 g/mL, the refractive index of 1.483 ± 0.01 and a specific rotation of $\alpha_{D}^{20}$ of +17.15 ± 1.92 (*c* 11.45, *C*H_2_Cl_2_*)*. The overall yield obtained for this plant was higher in comparison to the EO of *L. heteromorpha* with a value of 0.06% w/w as reported by Gilardoni et al. (2018), and much lower in comparison to *L. paniculata* with 0.49% w/w as reported by Panamito et al. (2021) and 0.317% w/w for *L. mutica* as reported by Ramírez et al. (2018).

### Chemical composition

**Figure 1.**
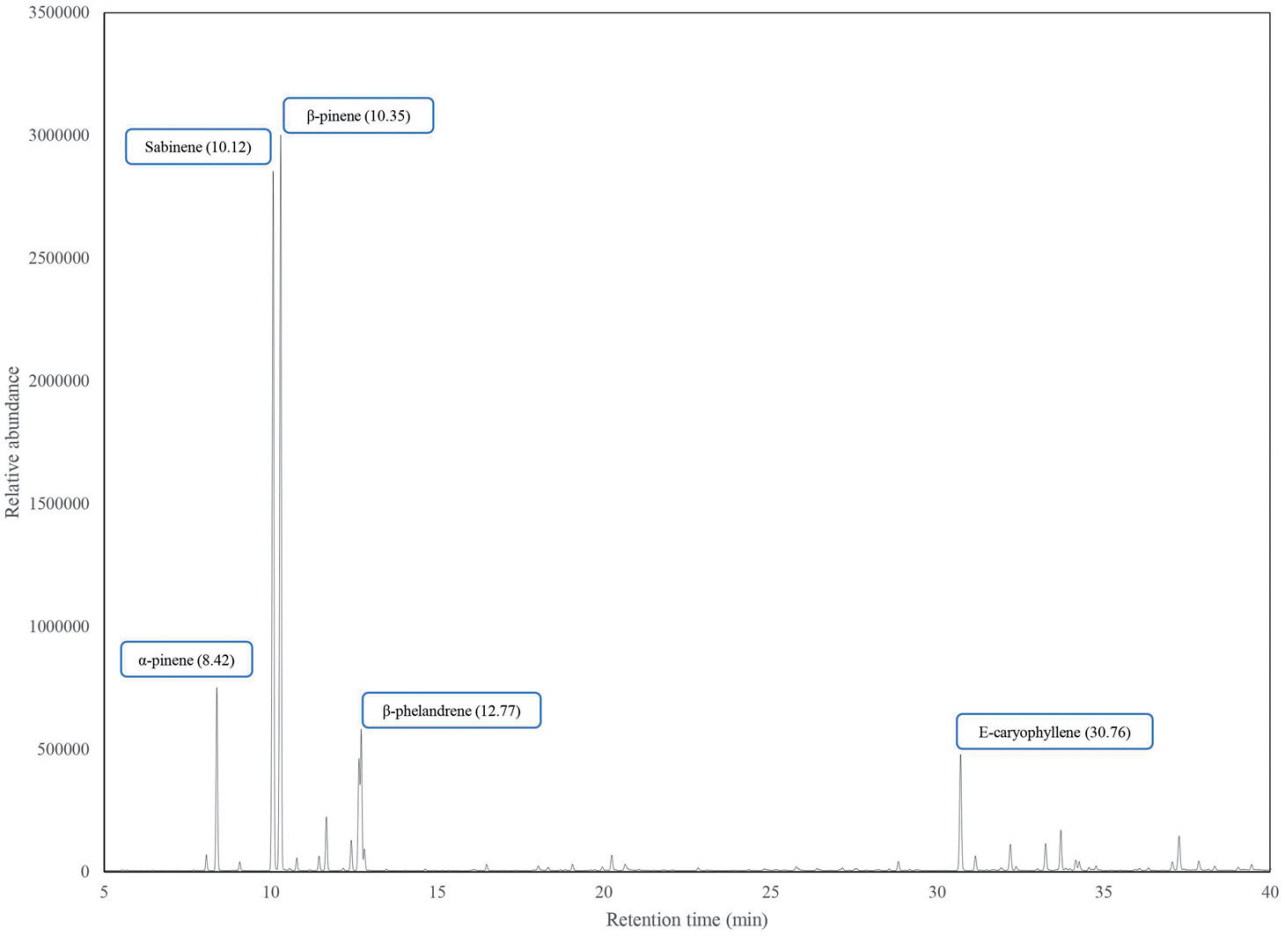
Chromatogram of the essential oil of *L. betonicifolia* from Ecuador.

A total of 39 components of the essential oil were identified, representing 97.55% of the total oil composition. The main compounds in *L*. *betonicifolia* EO from Ecuador were: β-pinene (30.45%), sabinene (27.98%), α-pinene (4.97%), β-phellandrene (4.79%), *E*-caryophyllene (4.44%) and limonene (3.84%). Monoterpene and sesquiterpene hydrocarbons were found to be the most representative chemical groups of the total oil composition with 76.51% and 16.71%, respectively. *L. betonicifolia* presents a distinctive leaf odour, mainly woody-herbaceous and citric but also a moderate spicy odour that could be attributed to the occurrence of α- and β-pinene, sabinene, β-phellandrene and limonene (Table 1, Figure 1). A related Ecuadorian species, *L. mutica*, was determined to present a woody, herbaceous and earthy odour due to the occurrence of α-pinene, β-phellandrene and dauca-5,8-diene, respectively, as determined by the AEDA analysis (Ramírez et al. 2017).

**Table 1.** Chemical composition of the essential oil of *L*. *betonicifolia* in DB-5MS.

Rt^a^	Compound	LRI^b^	LRI^c^	%	Σ
8.08	α-Thujene	924	924	0.35	0.12
8.39	α-Pinene	932	932	4.97	1.32
9.08	Camphene	948	946	0.30	0.04
10.08	Sabinene	971	969	27.98	2.94
10.30	*β*-Pinene	976	974	30.45	2.25
10.79	Myrcene	988	988	0.34	0.10
11.46	*p*-Mentha-1(7),8-diene	1003	1003	0.48	0.03
11.57	α-Phellandrene	1006	1002	0.28	0.31
11.68	delta-3-Carene	1008	1008	1.08	1.20
12.07	α-Terpinene	1016	1014	0.37	0.48
12.43	*p*-Cymene	1023	1020	0.36	0.39
12.66	Limonene	1028	1024	3.84	0.72
12.72	β-Phellandrene	1029	1025	4.79	2.16
12.83	1,8-Cineole	1032	1026	0.79	0.27
13.48	(*E*)-*β*-Ocimene	1045	1044	0.18	0.17
14.04	γ-Terpinene	1057	1054	0.22	0.19
14.66	*p*-Mentha-2,4,8 diene	1070	1085	0.38	0.52
15.34	Terpinolene	1084	1086	0.13	0.04
18.35	Camphor	1146	1141	0.20	0.06
19.07	Pinocarvone	1161	1160	0.47	0.53
19.96	Terpinen-4-ol	1179	1174	0.39	0.14
20.66	Myrtenal	1194	1195	0.73	0.80
28.84	α-Copaene	1374	1374	0.67	0.66
29.18	*β*-Bourbonene	1381	1387	0.19	0.23
30.70	(*E*)-Caryophyllene	1417	1417	4.44	0.91
31.15	*β*-Gurjunene	1427	1431	0.62	0.11
31.91	Aromadendrene	1445	1439	0.26	0.21
32.20	α-Humulen	1452	1452	0.85	0.44
32.37	allo-Aromadendrene	1456	1458	0.41	0.30
33.26	γ-Muurolene	1477	1478	2.84	2.68
33.71	γ-Curcumene	1488	1481	2.95	2.41
33.85	Bicyclogermacrene	1491	1500	0.50	0.36
34.16	Viridiflorene	1498	1496	2.11	1.94
34.56	γ-Cadinene	1508	1513	0.21	0.11
34.77	δ-Cadinene	1514	1522	0.43	0.18
36.34	*β*-Germacrene	1554	1559	0.25	0.14
37.27	Caryophyllene oxide	1577	1582	0.76	0.69
37.86	Guaiol	1592	1600	0.98	0.50
	Monoterpene hydrocarbons			76.51	
	Oxygenated monoterpene			2.58	
	Sesquiterpene hydrocarbons			16.71	
	Oxygenated sesquiterpene			1.74	
	Total amount of compounds			97.55	

^a^Rt: Retention time; ^b^LRI: calculated linear retention indices obtained on DB-5ms column using a series of *n*-alkanes (C9–C24) from [19]; ^c^LRI: reference linear retention indices from [20]; %: relative percentage amount.

This is the first report of the enantiomeric distribution of the EO, obtained from the aerial parts of *L*. *betonicifolia* from Ecuador. According to Caballero-Gallardo et al. (2011), a variation in the chemical composition for *L*. *betonicifolia* collected in Bucaramanga (Colombia), was detected by the occurrence of limonene (27.5%), α-pinene (19.4%), β-pinene (9.5%) and *trans*-β-caryophyllene (6.8%) as the main compounds, meanwhile, from our EO, limonene was one of the less abundant compounds with 3.84%, besides, the percentages of α-pinene and β-pinene varied greatly from these two EOs (Table 1). This can be explained by the fact that chemical composition can vary not only from the seasonal station but also, from countries in the same Andes region.

Studies on the chemical profiling of the EOs of species from the same genus report similar results to those presented in this study. Ruiz et al. (2015) reported the identification of α-pinene (29.87%), eucalyptol (13.25%) and β-pinene (9.64%) as majority compounds from the EO of *L. meyenii*. Likewise, in the essential oil of the species *L. mutica* the components are constituted mainly by monoterpene hydrocarbons (72.3%), and sesquiterpene hydrocarbons (15.4%) (Malagón et al. 2003). Similarly, Valarezo et al. (2012) in their study about the EO from *L. paniculata,* reported the occurrence of *δ*-3-carene (19.9%), β-pinene (17.01%) and β-phellandrene (8.6%) as main constituents and a composition mostly of monoterpene hydrocarbons (49.6%) followed by sesquiterpene hydrocarbons (37.1%). Morocho et al. (2017) reported for the EO of *L. radula* (Benth) Epling to the identification of δ-3-carene (19.9%), β-pinene (17.0%) and β-phellandrene as main compounds (8.6%). However, the chemical composition of the EO from related species can vary greatly as in the study of *L. graveolens* (Regel) Epling, where the main reported compounds were β-caryophyllene, *δ*-cadinene, and α-humulene (Arze et al. 2009) or in *L. floribunda* (Benth) Epling where the majority components were borneol, β-caryophyllene and ledyl acetate Velasco-Negueruela et al. (1994).

### Enantiomeric distribution

Chiral GC-MS analysis was performed to evaluate the enantiomeric distribution of the three monoterpenes and one sesquiterpene present in *L. betonicifolia* essential oil. In the EO analysed is remarkable the highest purity of the two enantiomers: (–)-β- pinene and (+)-sabinene (>87% e.e.) (Table 2).

**Table 2.** Enantioselective analysis of *L. betonicifolia* essential oil.

LRI^a^	Compound	Enantiomeric distribution %	(e.e)%^b^
931	α-Thujene +	61.72	23.44
933	α-Thujene −	38.28	
966	β- Pinene +	3.37	93.25
971	β- Pinene −	96.63	
988	Sabinene +	93.56	87.13
1000	Sabinene −	6.44	
1466	γ-Muurolene +	37.37	25.25
1473	γ-Muurolene −	62.63	

**^a^**LRI, Linear retention index calculated on the chiral capillary column diethyl tertbuthysilyl-β-cyclodextrin; **^b^**enantiomeric excess.

In our study, we report the identification of four pairs of enantiomers, in contrast to the enantiomeric study carried out by Ramírez et al. (2017). where 11 pairs of enantiomers and two pure enantiomers: (1 *R*)-(+)-camphor,. and (–)-(2 *R*)-borneol both with >99% e.e were reported. In the same report, (–)-(1*S*)-β-pinene, (1*S*,4*R*)-(–)-camphene, (*S*)-(+)-α-phellandrene and (*S*)-(+)-β-phellandrene were identified with an e.e. about 80 to 96.96% and (+) sabinene with a low purity (34.06% e.e.).

### Anticholinesterase activity

**Figure 2.**
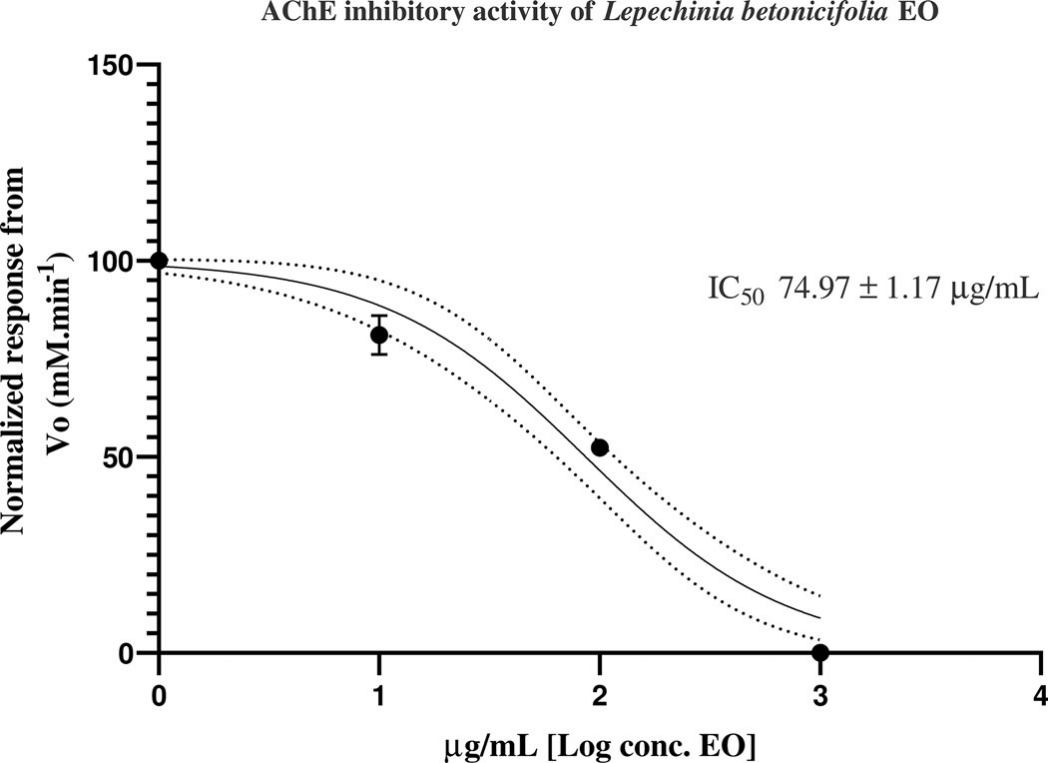
AChE inhibitory response from the essential oil of *L. betonicifolia* expressed as IC_50_, calculated from nonlinear regression curve data fitting analysis, *n* = 9.

The *L*. *betonicifolia* EO was assayed for its anticholinesterase potential by measuring the rate of reaction of AChE against three different concentrations of the essential oil. The IC_50_ value obtained for the EO was 74.97 ± 1.17 µg/mL, which can be considered to be a strong inhibitory effect (Figure 2). EO are considered strong due to their lipophilicity and small molecular size constituents (Phrompittayarat et al. 2014). Donepezil hydrochloride was used as a positive control with an IC_50_ value of 13.80 ± 1.01 nM (Valarezo et al. 2021).

To the best of our knowledge, this is the first report of the enantiomeric distribution and inhibitory effect of the EO of *L. betonicifolia* over the AChE enzyme. A similar study was conducted on a related species from the south of Ecuador, *Lepechinia paniculata*, where it was reported a strong inhibitory effect over the AChE enzyme with an IC_50_ value of 28.2 ± 1.8 µg/mL (Panamito et al. 2021). A weak inhibitory effect over AChE was observed for another related species from Argentina, *L*. *floribunda*, where a 33.4 ± 5.3 inhibition percentage at 10 mg/mL dose of the organic extract was reported (Carpinella et al. 2010).

Few studies of *Lepechinia* species showing cholinesterase activity have been conducted, among them, *L. paniculata* showed IC_50_ values of 28.2 ± 1.8 2 µg/mL (Panamito et al. 2021). According to the study conducted by Miyazawa and Yamafuji (2005) analysing the inhibitory potency of 17 kinds of bicyclic monoterpenoids, the (+) and (–)-α-pinene enantiomers showed moderate inhibitory activity against AChE with an IC_50_ value of 0.4 and 0.44 mM, respectively, meanwhile, the (–)-β-pinene isomer exerted a low inhibition (48.5%) at 1.0 mM dose. For sabinene, an IC_50_ value of 176.5 µg/mL against AChE was determined by Bonesi et al. (2010). In our work, sabinene and β-pinene account for more than 57% of the chemical composition of the EO, and from the enantiomeric analysis we can determine that the main enantiomers are (–)-β-pinene with >96% e.e. and (+) sabinene with 93.56% e.e. which can explain the fact that the EO exert a good inhibitory activity against AChE. Further studies should be conducted to determine the inhibitory profile of the enantiomers found in this study against AChE as well as their kinetics mechanisms. Monoterpenoids should be assessed as individual compounds or as a mixture as presented in the essential oil to characterise completely the inhibitory effect observed over AChE. Despite the low yield obtained for the EO, the identification of the inhibitory profile of the compounds or the mixture as they are occurring in this EO could provide the information to propose a formulation as adjuvant therapy in the treatment of neurodegenerative diseases such as Alzheimer’s disease.

To obtain a deeper understanding of the inhibitory effect of the compounds occurring in this medicinal species, polar solvent extractions can be made, according to the traditional mode of use. As it was mentioned before, the plant can be used as an infusion and it is possible to find phenolic compounds. Current research has confirmed the tight relationship between the generation of reactive oxygen species (ROS) and free radicals, and the development of cancer, diabetes, cardiovascular diseases and neurodegenerative disorders including Parkinson’s and Alzheimer’s diseases. Cellular redox imbalance could be corrected by the use of diverse polyphenolic nature compounds like flavonoids, phenolics, anthocyanins and they could be used as effective and safe Alzheimer’s disease therapy (Li et al. 2014).

## Conclusions

A complete characterisation of the chemical profiling, enantiomeric distribution, physical properties and anticholinesterase effect was made for the essential oil from the leaves of *Lepechinia betonicifiolia*. More than 50% of the chemical composition is due to the occurrence of β-pinene and sabinene. Four pairs of enantiomers were detected and identified. The anticholinesterase effects presented here encourage us to propose extensive studies to determine the compounds responsible for the inhibitory effect and the kinetic mechanisms exerted against AChE in order to include this EO or their components in a natural pharmaceutical formulation as adjuvant therapy for Alzheimer’s disease. Several EO from *Lepechinia* species have been studied so far to date with interesting inhibitory effects over cholinesterase enzymes but non-volatile fraction needs to be further analysed to find a better application of these medicinal species in the drug discovery field. The anti-inflammatory and antioxidant effects of the EO can be further analysed in order to determine a close relationship with the anticholinesterase effect observed.
